# Adverse Skin Reactions to Personal Protective Equipment Among Healthcare Workers in Oman During the Coronavirus Disease 2019 Pandemic

**DOI:** 10.7759/cureus.33223

**Published:** 2023-01-01

**Authors:** Faisal Al Badri, Aisha Al Ali, Yaqoub Al Saidi, Zeyana Al Bahri, Saud Al Hashimi

**Affiliations:** 1 Occupational Medicine, Armed Forces Hospital, Muscat, OMN; 2 Dermatology, Al Nahdha Hospital, Muscat, OMN; 3 Family Medicine and Public Health, Sultan Qaboos University Hospital, Muscat, OMN; 4 Dermatology, Armed Forces Hospital, Muscat, OMN

**Keywords:** surgical mask, allergic dermatitis, covid 19, health care workers, personal protective equipment, occupational contact dermatitis

## Abstract

Background

During the coronavirus disease 2019 (COVID-19) pandemic, healthcare workers (HCWs) were required to use personal protective equipment (PPE) for unusually prolonged periods of time in order to protect themselves. This study was conducted to assess the prevalence of adverse skin reactions to PPE among HCWs from occupational and domestic exposure in Oman.

Methods

This was a cross-sectional study that used a self-administered questionnaire, modified based on the Nordic Occupational Skin Questionnaire, and was conducted in different categories of healthcare facilities in Oman from September to December 2020. This study involved 431 different categories of HCWs. Stata statistical software, version 12 (StataCorp, College Station, TX), was used to analyze the data, with a P value <0.05 indicating statistical significance.

Results

Findings indicated that 58.24% of HCWs reported new skin symptoms since the pandemic started, compared to 33.41% of HCWs who had skin symptoms before the pandemic (P<0.001). From the multivariate analysis, being female (odds ratio, or OR, 3.512; 95% confidence interval, or CI: 2.193-5.625), allergic rhinitis diagnosis (OR 2.420; 95% CI: 1.097-5.347), history of skin symptoms (OR 3.166; 95% CI: 1.856-5.400), and total glove use time (OR 1.160; 95% CI: 1.078-1.247) were associated with an increased risk of acquiring new skin symptoms.

Conclusion

This study demonstrates that there is some association between the prolonged use of PPE during an event such as a pandemic and a previous history of allergic rhinitis and skin symptoms. This study also emphasizes the importance of appropriate protective skin care before and after the use of PPE.

## Introduction

The rate of occupational skin conditions increased, particularly for healthcare workers (HCWs), during the coronavirus disease 2019 (COVID-19) pandemic [1]. Due to the nature of HCWs’ duties, they are required to use personal protective equipment (PPE) for unusually prolonged periods of time, in order to protect themselves from contracting infections as well as to control the transmission of diseases, such as COVID-19. Examples of PPE used during the COVID-19 pandemic are N95 masks, surgical masks, gloves, gowns, and goggles [2].

Although PPE is used to protect HCWs from infections, it can cause various adverse skin reactions due to prolonged use [3]. Prior to the COVID-19 pandemic, the estimated prevalence of occupational skin diseases in HCWs ranged from 16.5% to 55%, depending on the country and reporting system used [4]. However, after the pandemic, occupational skin lesions were reported in up to 97% of HCWs, due to the strict protection measures, mainly affecting the nasal bridge, cheek, forehead, and hands [5]. According to data from Wuhan, the prevalence of skin reactions has increased: out of 376 responders, 74% experienced adverse skin reactions in a general survey. Similar reports from China showed 49.0% of HCWs who responded to an online survey reported mask-related facial skin reactions following an unusual prolonged use of N95 and surgical masks [6]. A study in Saudi Arabia found that the overall prevalence of self-reported skin conditions during the COVID-19 pandemic was 46.3% [7]. To our knowledge, there has been no previous study conducted in Oman to evaluate occupational skin conditions among HCWs.

The risk factors for hand dermatitis among HCWs include atopy, winter season, low humidity, frequency of hand washing, wet work, glove use, and duration of employment [1]. The duration of exposure appears to be the leading risk factor for face dermatitis; particularly, masks and goggles worn for over six hours and hand washing more than 10 times a day leads to an increased risk of skin damage [8]. Moreover, additional exposure to PPE outside work, such as using protective measures in public places, might increase the risk of developing adverse skin reactions. To the best of our knowledge, no prior study has evaluated both domestic and workplace PPE exposure. This study aimed to assess the prevalence of occupational skin diseases and adverse skin reactions among HCWs in Oman, as well as the risk factors for developing adverse skin reactions to PPE, considering occupational and domestic use. We hypothesized that there is an increased risk of adverse skin reactions among HCWs because of the prolonged workplace and domestic PPE use. Our second hypothesis was that the prolonged use of PPE and detergents during the COVID-19 response increases the prevalence of occupational skin diseases and adverse reactions among HCWs. The last hypothesis is that a previous medical history of skin disease or allergic conditions increases the risk of adverse skin reactions.

## Materials and methods

Study design, population and sampling

This is a cross-sectional study that was conducted from September to December 2020 among all categories of HCWs, that is, doctors, nurses, dentists, dental assistants, pharmacists, laboratory technicians and medical orderlies, in Oman. All categories of health facilities were also included in the study, such as hospitals, polyclinics, and health centers. The estimated prevalence range of occupational skin diseases in HCWs is 16.5%-55% [4]. The assumed prevalence of occupational skin diseases among HCWs in Oman is the average of the above-mentioned range, which is approximately 36%. Using this information, the minimum sample size for estimating the prevalence range of occupational skin diseases among HCWs in Oman within 5% of the true value with 95% confidence was 355.

A convenience sampling method was used in this study. Data was collected from available respondents who filled out the questionnaire. The total number of participants was 431.

Study instrument

An online self-administered modified questionnaire based on the Nordic Occupational Skin Questionnaire (NOSQ-2002) was sent through a link to all accessible HCWs in Oman [9]. Also, the questionnaire was distributed and filled out manually in three big hospitals in the Muscat governorate: Al Nahda Hospital, Sultan Qaboos University Hospital and Armed Forces Hospital.

The questionnaire gathered information on acute and chronic dermatological symptoms prior to the COVID-19 pandemic and their potential to be work related. It also covered information related to new skin symptoms that developed after March 2020 and any prior skin conditions that worsened during the COVID-19 pandemic, and their potential to be work related. Furthermore, the questionnaire gathered information regarding the intensity of workplace and domestic exposure to PPE.

Data management and analysis plan

Stata statistical package (version 12) was used to analyze the data. Exploratory data analysis was carried to check for the presence of outliers, the extent of missing data, as well as the distribution of the key variables and any transformation needed. Contingency tables were used for bivariate analysis to examine associations between outcome variables (adverse skin reactions) and covariates, and the chi-square (χ^2^) test was used for statistical inference. All statistically significant covariates in the bivariate analyses were included in the multivariable analysis. Multivariable logistic regression was used to estimate adjusted odds ratios (adj. ORs) and 95% confidence intervals (CIs). In all of the analyses, P<0.05 was regarded as statistically significant.

## Results

Socio-demographic characteristics

The total number of HCWs who responded to the invitation was 431; their socio-demographic characteristics are presented in Table 1. The mean age was 37.30 years (±7.74). Most participants were female (65.89%), doctors (48.03%) and nurses (32.71%). Among the participants, 16.47% were working in COVID-19 triage and isolation rooms. Around 35% of participants had been diagnosed with skin and/or non-skin allergies, and 33.41% had one or more facial and/or hand skin symptoms prior to the pandemic.

**Table 1:** Socio-demographic characteristics of HCWs who participated in the study COVID-19, coronavirus disease 2019; HCW, healthcare worker; HC, health center *Mean ± SD

Predictor		n (%)
Age (years)*		37.30 ± 7.74
Gender (female)		284 (65.89%)
Nationality (Omani)		288 (66.82%)
Job	Doctor	207 (48.03%)
	Nurse	141 (32.71%)
	Other	83 (19.26%)
Total service years*		13.52 ± 8.46
Department	Exclusively outpatient	134 (31.09%)
	Multiple departments including COVID-19 triage clinic and isolation wards	71(16.47%)
	Multiple departments not including COVID-19 triage clinic and isolation wards	56 (12.99%)
	Others	170 (39.44%)
Facilities	Hospital	283 (65.66%)
	Polyclinics	58 (13.46%)
	Local HC	79 (18.33%)
	Others	11 (2.55%)
Diagnosed with allergic disease before the pandemic	No	280 (64.97%)
	Atopic dermatitis or eczema	34 (7.89%)
	Urticaria	13 (3.02%)
	Non-skin allergy	104 (24.13%)
Any facial or hand skin symptoms before the pandemic	No	287 (66.59%)
	Yes	144 (33.41%)

Exposure to PPE

Table 2 presents exposure characteristics and the self-reported skin adverse effects. Most of HCWs (53.83%) used multiple types of eye and facial PPE at work, while the majority (86.31%) used strictly surgical face masks outside work. At work, HCWs used facial and eye PPE for 7.47 hours (±7.06), compared to 2.47 hours (±2.16) outside work. Around 90% of HCWs used gloves at work for 3.68 hours (±2.91), compared to around 31% of HCWs who used gloves outside work for 0.53 hours (±1.44). More than 99% of HCWs were washing hands at work, at an average of 12.34 times/day (±10.76), and more than 99% were using hand sanitizers at work an average of 15.47 times/day (±12.66). More than 98% of HCWs were washing hands outside work, with an average of 9.63 times/day (±7.65), and more than 71% were using hand sanitizers outside work with an average of 4.34 times/day (±6.65). The majority of HCWs were using hand moisturizers after hand washing either regularly (28.77%) or occasionally (39.91%).

**Table 2:** Exposure characteristics and skin adverse outcomes in HCWs HHW, healthcare worker; PPE, personal protective equipment *Mean ± SD

Predictor			n (%)
Face and eye PPE used at work since the pandemic started	None		7 (1.62%)
	Strictly medical/surgical mask		186 (43.16%)
	Strictly N95 mask		6 (1.39%)
	Strictly face shield		0 (0%)
	Strictly goggles		0 (0%)
	Multiple		232 (53.83%)
Face and eye PPE used outside work since the pandemic started	None		15 (3.48%)
	Strictly medical/surgical mask		372 (86.31%)
	Strictly N95 mask		5 (1.16%)
	Strictly face shield		3 (0.70%)
	Strictly goggles		1 (0.23%)
	Multiple		31 (7.19%)
	Others		4 (0.93%)
Face and eye PPE usage time at work (hours)*			7.47±7.06
Face and eye PPE usage time outside work (hours)*			2.47±2.16
"New" facial symptoms since the pandemic started	No		225 (52.20%)
	Rash		18 (4.18%)
	Redness or erythema		7 (1.62%)
	Itch		16 (3.71)
	Dry or desquamated skin		8 (1.86%)
	Others		23 (5.34%)
	Multiple symptoms		134 (31.09%)
Gloves used at work since the pandemic started	None		42 (9.74%)
	Natural rubber/latex		207 (48.03%)
	Synthetic rubber (e.g. nitrile, neoprene, etc.)		37 (8.58%)
	Plastic (e.g. vinyl, PVC, polyethene)		22 (5.10%)
	Multiple types		83 (19.26%)
	Not known		40 (9.28%)
Gloves used outside since the pandemic started	None		296 (68.68%)
	Natural rubber/latex		55 (12.76%)
	Synthetic rubber (e.g. nitrile, neoprene, etc.)		7 (1.62%)
	Plastic (e.g. vinyl, PVC, polyethene)		36 (8.35%)
	Multiple types		12 (2.78%)
	Not known		25 (5.80%)
Gloves usage time at work (hours)*			3.68±2.91
Gloves usage time outside work (hours)*			0.53±1.44
Washing hands at work		No	3 (0.71%)
		Yes	420 (99.29%)
Washing hands outside work		No	6 (1.41%)
		Yes	421 (98.59%)
Washing hand at work (times)*			12.34±10.76
Washing hand outside work (times)*			9.63±7.65
Sanitizing hand at work		No	3 (0.71%)
		Yes	419 (99.29%)
Sanitizing hand outside work		No	123 (28.81%)
		Yes	304 (71.19%)
Sanitizing hand at work (times)*			15.47±12.66
Sanitizing hand outside work (times)*			4.34±6.65
Using moisturizer after hand washing	No		135 (31.32%)
	Yes		124 (28.77%)
	Occasional		172 (39.91%)
"New" hand symptoms since the pandemic started	No		268 (62.33%)
	Rash		3 (0.70%)
	Redness or erythema		4 (0.93%)
	Itch		13 (3.02%)
	Dry or desquamated skin		61 (14.19%)
	Others		3 (0.70%)
	Multiple symptoms		78 (18.14%)
Any "new" facial or hand skin symptoms since the pandemic started	No		180 (41.76%)
	Yes		251 (58.24%)

Self-reported skin adverse symptoms

There were 251 (58.24%) HCWs who reported new facial and/or hand skin symptoms since the pandemic started (47.8%, new facial skin symptoms; 37.67%, new hand skin symptoms) compared to 144 HCWs who had facial and/or hand skin symptoms prior to the pandemic (P<0.001). Rashes were the most reported single facial symptom, while dryness was the most reported hand symptom.

In univariate analysis, gender, age, years of service, being diagnosed with allergic rhinitis or multiple allergic diseases, complaining of naso-ocular symptoms, or any facial or hand skin symptoms before the pandemic and total time of glove use (at work and outside work) were significantly associated with new facial and/or hand skin symptoms (Table 3). In the multivariate analysis, being female (OR 3.512; 95% CI: 2.193-5.625), years of service (OR 0.973; 95% CI: 0.948-0.999), being diagnosed with allergic rhinitis (OR 2.420; 95% CI: 1.097-5.347), complaining of any facial or hand skin symptoms prior to the pandemic (OR 3.166; 95% CI: 1.856-5.400), and total glove use time (OR 1.160; 95% CI: 1.078-1.247) were associated with an increased risk of getting new facial and/or hand skin symptoms (Table 4).

**Table 3:** Determinants of new facial and hand symptoms among HCWs in univariate regression models HCW, healthcare worker; OR, odds ratio

Predictor		OR	95% confidence interval	P value
Gender (female)		3.404	2.247-5.156	<0.001
Age		0.964	0.940-0.989	0.005
Total service years		0.974	0.950-0.998	0.033
Established diagnosis of allergic disease	Allergic rhinitis (hay fever)	2.227	1.122-4.422	0.022
	Allergic conjunctivitis	1.810	0.444-7.379	0.408
	Asthma	1.056	0.346-3.220	0.924
	Food allergy	0.905	0.256-3.195	0.876
	Atopic dermatitis/eczema	3.317	0.906-12.148	0.070
	Urticaria	0.565	0.181-1.771	0.328
	Multiple without atopic dermatitis/eczema	3.167	1.240-8.083	0.016
	Multiple with atopic dermatitis/eczema	5.12	1.470-17.888	0.010
Chest symptoms		1.217	0.622-2.380	0.566
Nasocular symptoms		2.287	1.357-3.857	0.002
Any facial or hand skin symptoms before the pandemic		3.727	2.362-5.880	<0.001
Type of mask used at work	Medical/surgical mask	1.333	0.290-6.122	0.711
	N95 mask	6.667	0.487-91.331	0.155
	Multiple types	2.439	0.533-11.162	0.251
Type of mask used outside work	Medical/surgical mask	0.692	0.232-2.065	0.510
	N95 mask	0.75	0.093-6.604	0.787
	Multiple types	0.607	0.168-2.196	0.447
Total time of mask use		1.015	0.978-1.054	0.423
Type of gloves used at work	Natural rubber/latex	1.634	0.837-3.193	0.150
	Synthetic rubber (e.g. nitrile, neoprene, etc.)	1.569	0.644-3.819	0.321
	Plastic (e.g. vinyl, PVC, polyethene)	2.857	0.965-8.460	0.058
	Multiple types	3.278	1.512-7.106	0.003
Type of gloves used outside work	Natural rubber/latex	1.068	0.598-1.908	0.823
	Synthetic rubber (e.g. nitrile, neoprene, etc.)	4.963	0.590-41.735	0.140
	Plastic (e.g. vinyl, PVC, polyethene)	2.481	1.128-5.459	0.024
	Multiple types	4.135	0.891-19.202	0.70
Total time of glove use		1.108	1.044-1.175	0.001
Total hand wash		1.005	0.993-1.019	0.376
Total hand sanitizer		1.003	0.991-1.016	0.627

**Table 4:** Determinants of new facial and hand symptoms among HCWs in the multivariate regression model HCW, healthcare worker; OR, odds ratio

Predictor		OR	95% confidence interval	P value
Gender (female)		3.512	2.193-5.625	<0.001
Total service years		0.973	0.948-0.999	0.044
Established diagnosis of allergic disease	Allergic rhinitis (hay fever)	2.42	1.097-5.347	0.029
	Allergic conjunctivitis	1.541	0.290-8.178	0.611
	Asthma	0.775	0.232-2.586	0.678
	Food allergy	0.892	0.216-3.686	0.874
	Atopic dermatitis/eczema	2.249	0.533-9.495	0.270
	Urticaria	0.283	0.077-1.041	0.058
	Multiple without atopic dermatitis/eczema	2.326	0.824-6.562	0.111
	Multiple with atopic dermatitis/eczema	2.534	0.657-9.776	0.177
Any facial or hand skin symptoms before the pandemic		3.166	1.856-5.400	<0.001
Total time of mask use		0.979	0.944-1.016	0.254
Total time of glove use		1.160	1.078-1.247	<0.001
Total hand wash and sanitizer use		1.00	0.991-1.008	0.922

## Discussion

To the best of our knowledge, this is the only report to date that has focused on occupational and domestic exposure to PPE and the development of adverse skin reactions in the same individuals. Most of the previous studies reported the adverse skin reactions to PPE either from occupational exposure [1,5,7,10] or public exposure [11,12].

The majority of participants were female (n = 284, 65.89%), which is comparable with some other studies [7,10]. Around 35% of participants had been diagnosed with skin and/or non-skin allergies, and 33.41% had one or more of facial and/or hand skin symptoms before the pandemic started, which is nearly comparable with another study that found the incidence of occupational skin disease to range from 16.5% to 55% [4].

Exposure characteristics

Since the pandemic started, HCWs have been using PPE for the face, eyes and hands more frequently and for longer time periods. With regard to domestic use of face PPE, the majority of HCWs (86.31%) were using strictly medical/surgical masks alone outside work for around 2.47 hours (±2.16). There were few studies conducted focusing on the domestic use of PPE. Among these was a study that was carried out in China, including 20 healthy participants who had used N95 masks and surgical/medical masks. The total duration of mask wearing was six hours for each participant [12]. Another larger study focused on the domestic use of face masks found that 46% of participants were using cloth masks, followed by 37% using surgical masks and only 17% wearing N95 masks. The majority of those participants (58%) were wearing masks for less than six hours, whereas 35% used the masks for 6-12 hours. This is comparable with the current study population, where most of participants used the mask outside of work for less than six hours [11]. With regard to the domestic use of hand PPE, we could not find another study for comparison.

When it comes to occupational exposure, most of HCWs used multiple types of face and eye PPE (53.83%), whereas 43.16% used strictly medical/surgical masks for long periods of time: 7.47 hours (±7.06). Some reports found that majority of HCWs were wearing N95 masks during the pandemic on a regular basis, which was not significantly observed in the current study [13]. This could be due to the shortage of N95 masks in Oman at the beginning of the pandemic.

Around 90% of HCWs used gloves at work for 3.68 hours (±2.91); 48.03% of HCWs were using purely natural rubber/latex gloves whereas 19.26% were using different types of gloves. This is comparable to other published papers that reported that the majority of HCWs were using mainly latex gloves [3,8]. More than 99% of HCWs were washing hands at work an average of 12.34 times/day (±10.76), and more than 99% were using hand sanitizers at work an average of 15.47 times/day (±12.66). Similar results were observed in other studies, with the majority of HCWs practicing hand hygiene more than 10 times per day [5,10].

Skin adverse outcomes

There were 251 (58.24%) HCWs who reported new facial and/or hand skin symptoms since the pandemic started compared to 144 (33.41%) HCWs who had facial and/or hand skin symptoms prior to the pandemic (P<0.001). After the pandemic, 47.8% reported one or more new facial skin symptoms, and 37.67% reported one or more new hand skin symptoms. Rashes were the most common single reported facial symptom, while dryness was the most common single reported hand symptom.

This might be explained by the huge exposure and prolonged duration of wearing PPE during the pandemic along with the more frequent use of detergents and disinfectants. All these factors can result in friction and disruption of the skin barrier and hyper-hydration that predisposes the skin to irritation or exacerbation of pre-existing dermatosis [1,14].

The prevalence of new facial symptoms since the pandemic started was reported in 47.80% of the sample in the current study, compared to 74.5% in a study conducted in China [10]. In the current study, the face was the most commonly affected site compared to the hands, as the majority of participants were using face mask inside and outside of work compared to gloves, with contact times for the face mask being longer than that for gloves. This finding is consistent with another published paper that found that the face was affected more than the hands [5]. However, a systematic review showed that the nasal bridge was more affected due to pressure, especially with the N95 masks, followed by hand dermatitis from PPE, followed by cheeks and other parts of head and neck [15].

In the current study, 31.09% of participants reported multiple new facial symptoms after the pandemic started; however, rashes were the most reported single facial symptom in 18 participants (4.18%) followed by itch, dryness, desquamation of skin and erythema. In comparison to some other reports, dryness and itchiness were observed more often compared to other symptoms [7,10]. One of the most notable adverse reactions to face masks that has been reported in the past prior to the pandemic is contact dermatitis due to a variety of allergens present in the masks. This side effect requires special attention regarding the diagnosis and identification of the potential allergen, as well as management and prevention measures [4]. We could not confirm the presence of contact dermatitis as a cause of new facial symptoms as no patch tests were done on the participants.

New hand symptoms were reported since the pandemic in 37.67% of the participants in the current study, with 18.14% reporting multiple symptoms. However, as a single reported symptom, dryness was the most commonly observed adverse hand skin reaction (14.19%) followed by itch (3.02%), and this was comparable with another report [7]. It was expected that there would be a correlation between the development of new facial and hand skin symptoms and the exposure to PPE during the pandemic, proved in the cohort in this study to be statistically significant. However, in the context of having a higher percentage of participants who developed a new onset of facial symptoms, it was expected that there would be a significant correlation between the duration of face PPE use and the development of new facial symptoms, but that was not statistically significant, despite prolonged face mask use (7.47±7.06). This might be explained by the fact that most participants in our study used medical/surgical masks due to the severe shortage of N95 masks in the beginning of the pandemic, as mentioned previously. Most reported facial adverse effects were noted with N95 masks, which were mainly pressure related [6]. One study, conducted in China, where participants were exposed to both types of masks, noted more adverse skin reactions due to N95 masks compared to the medical/surgical masks [12].

Conversely, the correlation between the development of new hand symptoms and the duration of glove use was significant, with a P value of 0.001. This was not observed in another study that did not find a significant correlation between skin symptoms and the duration of glove wearing [5]. However, the proportion of HCWs who suffered from new onset hand symptoms was lower than those who suffered from new facial symptoms, which is the opposite to another study where the hands were the most commonly affected area [7].

A significant correlation was not noted in this study between the frequency of hand washing, use of detergents and sanitizers and the development of adverse skin reactions, the opposite to what was predicted, although these were among the most recognized triggers for skin reactions among the HCWs. This significant correlation with hand hygiene and development of skin reactions was noted in another paper [5]. The lower incidence of getting new hand symptoms might be explained through the regular or occasional use of hand moisturizers among the majority of the participants. Compliance with skin care protective measures can lead to less adverse skin reactions and a better adherence to PPE usage, a finding which has been noted in another published report [14].

In the univariate analysis, gender, age, years of service, being diagnosed with allergic rhinitis or multiple allergic diseases, complaining of naso-ocular symptoms, any facial or hand skin symptoms prior to the pandemic and total time of glove use (at work and outside work) were significantly associated with new facial and/or hand skin symptoms. It was observed in the multivariate analysis that the main predictors for developing adverse skin reactions to PPE among HCWs were being female (P<0.001), pre-existing facial and hand skin symptoms prior to the pandemic (P<0.001), total duration of glove use at work and outside work (P=0.001), total years of service (P=0.044) and an established diagnosis of allergic rhinitis (hay fever) prior to the the pandemic (P=0.022).

In the current literature, atopy, glove use and duration of employment have been noted to be important risk factors for hand dermatitis [1,16]. Pre-existing facial inflammatory dermatosis was reported as a risk factor for developing adverse facial skin reaction in another publication [6]. Being female was also reported as a risk factor in other studies [7,10]. Other reports also noted the association between wearing face and hand PPE for long periods of time and the development of adverse skin reactions [10,13,14].

To date, there have only been a few published papers that have studied the relationship between atopy and contact dermatitis. One of these papers found a significant correlation between the occurrence of occupational hand dermatitis and the presence of atopy (P<0.001). The atopic disorders that were studied included atopic dermatitis, atopic mucosal symptoms, atopic skin diathesis, and family atopy, although patch tests were not performed in this study and the diagnosis of hand dermatitis was based on clinical assessment [17]. Another study, conducted among young German participants, found a significant association between self-reported nickel allergies, incident wheezing, and incident asthma. However, there was no significant association between nickel allergy and incident rhinoconjunctivitis [18]. To the best of our knowledge, being diagnosed with allergic rhinitis was not reported before to be a risk factor for developing new adverse skin reactions or contact dermatitis.

One of the limitations of this study is that this was a survey-based study with self-perceived symptoms without confirmation by dermatologists. A thorough explanation of the signs and symptoms was given with each question. However, it would have been more accurate if one-to-one interviews were conducted while the survey was being filled in to avoid any misunderstandings. There is a risk of information bias as some candidates may have not understood the questions or options despite a pilot study being done prior to the study being conducted.

## Conclusions

To the best of our knowledge, this is the first study to address adverse skin reactions to PPE in Oman before and after the COVID-19 pandemic, and also the only study to cover both occupational and domestic PPE use in the same cohort. This report is significant in terms of increasing awareness about the potential adverse skin reactions to PPE for both HCWs and the community. It is also important in terms of emphasizing appropriate protective skin care before and after the use of PPE and managing different skin reactions when they occur, regardless of the protective measures used. This would lead to higher user satisfaction and compliance with the usage of PPE among HCWs, and the public as well.
